# Diversity in olfactory bulb size in birds reflects allometry, ecology, and phylogeny

**DOI:** 10.3389/fnana.2015.00102

**Published:** 2015-07-29

**Authors:** Jeremy R. Corfield, Kasandra Price, Andrew N. Iwaniuk, Cristian Gutierrez-Ibañez, Tim Birkhead, Douglas R. Wylie

**Affiliations:** ^1^Department of Psychology, University of Alberta, EdmontonAB, Canada; ^2^Department of Neuroscience, University of Lethbridge, LethbridgeAB, Canada; ^3^Lehrstuhl für Zoologie, Technische Universität MünchenFreising-Weihenstephan, Germany; ^4^Department of Animal and Plant Sciences, University of SheffieldSheffield, UK

**Keywords:** olfactory bulb, comparative neuroanatomy, olfaction, sensory ecology, avian ecology

## Abstract

The relative size of olfactory bulbs (OBs) is correlated with olfactory capabilities across vertebrates and is widely used to assess the relative importance of olfaction to a species’ ecology. In birds, variations in the relative size of OBs are correlated with some behaviors; however, the factors that have led to the high level of diversity seen in OB sizes across birds are still not well understood. In this study, we use the relative size of OBs as a neuroanatomical proxy for olfactory capabilities in 135 species of birds, representing 21 orders. We examine the scaling of OBs with brain size across avian orders, determine likely ancestral states and test for correlations between OB sizes and habitat, ecology, and behavior. The size of avian OBs varied with the size of the brain and this allometric relationship was for the most part isometric, although species did deviate from this trend. Large OBs were characteristic of more basal species and in more recently derived species the OBs were small. Living and foraging in a semi-aquatic environment was the strongest variable driving the evolution of large OBs in birds; olfaction may provide cues for navigation and foraging in this otherwise featureless environment. Some of the diversity in OB sizes was also undoubtedly due to differences in migratory behavior, foraging strategies and social structure. In summary, relative OB size in birds reflect allometry, phylogeny and behavior in ways that parallel that of other vertebrate classes. This provides comparative evidence that supports recent experimental studies into avian olfaction and suggests that olfaction is an important sensory modality for all avian species.

## Introduction

The importance of olfaction to bird ecology and behavior has largely been underplayed. In fact, early ornithologists debated if birds had a sense of smell at all (Bang and Cobb, 1968; Roper, 1999). However, recent interest in avian olfaction is demonstrating that birds have a fully functional olfactory system and some species rely heavily on their sense of smell for many tasks. (see reviews Roper, 1999; Hagelin, 2007; Caro and Balthazart, 2010; Castro et al., 2010). Birds use olfaction in a wide range of contexts that parallel that of mammals and other vertebrates, including: foraging (Stager, 1964; Wenzel, 1968; Grubb, 1972), predator avoidance (Amo et al., 2008, 2011; Roth et al., 2008; Zidar and Lovlie, 2012; Stanbury and Briskie, 2015), advertisement and mate selection (Balthazart and Schoffeniels, 1979; Balthazart and Taziaux, 2009; Caro and Balthazart, 2010; Amo et al., 2012; Whittaker et al., 2013; Caro et al., 2015), to discriminate conspecifics, consubspecifics, and sexes (Mihailova et al., 2014) and even for territorial scent marking (Castro et al., 2010). To support these behaviors, sensitivities to odors in birds are comparable to that of some mammals (Stattleman et al., 1975; Snyder and Peterson, 1979; Smith and Paselk, 1986; Walker et al., 1986; Waldvogel, 1989; Clark et al., 1993).

The primary neural structures of the vertebrate forebrain involved in olfaction are the multi-layered olfactory bulbs (OBs). OBs vary considerable in relative size across vertebrates and in mammals and fishes this variation is not driven by the size of the rest of the brain; there is a high level of allometric independence from the rest of the brain (Finlay and Darlington, 1995; Finlay et al., 2001; Reep et al., 2007; Gonzalez-Voyer et al., 2009; Yopak et al., 2010, 2015). Much of this variation is correlated with an animal’s behavioral ecology, especially those behaviors that require improved olfactory sensitivities. For example, larger OBs are found in nocturnal primates and insectivores, reflecting their increased reliance on olfaction for foraging and social interactions when vision is constrained under low light levels (Barton et al., 1995). In birds, differences in foraging ecology, habitat, nesting strategy, diet, and activity patterns have been correlated with variations in OB sizes (Bang, 1971; Bang and Wenzel, 1985; Healy and Guilford, 1990; Hagelin, 2004; Hammock, 2005; Mackay-Sim and Royet, 2006; Buschhuter et al., 2008; Corfield et al., 2014). This strong correlation between olfactory mediated behaviors and relative OB sizes across vertebrates has led to the suggestion that the size of OBs is a functional adaptation rather than a phylogenetic consequence (e.g., Healy and Guilford, 1990; Gittleman, 1991; Barton et al., 1995; Corfield et al., 2014; Yopak et al., 2015), although this has yet to be tested on a large scale among vertebrates (but see Yopak et al., 2010).

Although it is clear that variations in OB sizes in birds can be attributed to aspects of their behavioral ecology, there is also a trend for more basal species, such as Apterygiformes (kiwi), and Anseriformes (ducks), to have larger OBs (Bang and Cobb, 1968; Wenzel, 1971). This led to the conclusion that modern birds (neornithines) were descended from ancestors that had a heavy reliance on olfaction, and a shift away from olfaction resulted from visual and vestibular sensory enhancements associated with flight (Wenzel, 1971; Alonso et al., 2004; Milner and Walsh, 2009). Indeed, recent evidence suggests that OB sizes initially increased during non-avian maniraptoriform evolution and then further increased during basal bird and early neornithine evolution (Zelenitsky et al., 2011).

Together with phylogeny, ecology, and behavior, the rules that govern the scaling of OBs with brain/body size are also likely to be influencing the relative sizes of OBs. Indeed, the patterns of neural scaling can be an important determinant of the size of specific brain regions (Herculano-Houzel, 2009; Ribeiro et al., 2014; Corfield et al., 2015). Therefore, it is clear that many factors, including, but not limited to, phylogeny, ecology/behavior, and neural scaling patterns are driving the diversity in OB sizes in birds. However, the degree in which each of these variables has shaped the evolution of olfaction in birds in currently unknown.

Variations in the size of the OBs were first described in birds by Bang and Cobb (1968), who produced a data set of OB ratio percentages (OB diameter/hemisphere diameter) in 108 species, which was obtained by measuring the greatest diameter of the OBs and hemispheres. They showed that the OB ratios ranged from over 37 in snow petrels (*Pagodroma nivea*) to less than 5 in some Passeriformes, providing the first evidence that species difference in the size of OBs was functionally significant. However, as noted by Corfield et al. (2014) and Caro et al. (2015), the methods used in this study do not account for species differences in brain morphology and are unlikely to provide an accurate estimate of OB size. Bang and Cobb (1968) also note that there were difficulties and inaccuracies associated with measuring the “longest diameter” of organs that are not spherical. Although the OB measurements of Bang and Cobb (1968) have been used in several studies (Healy and Guilford, 1990; Zelenitsky et al., 2011), we developed a new data set using measurements from histological sections, allowing for a more accurate examination of OB sizes across birds and for more species.

Using this new data set, we examine the size of OBs in 135 species of birds, representing 21 different orders. We determine the scaling of the OBs with brain size, the effects of phylogeny, and diversification on OB sizes across a phylogeny and test for correlations of OB sizes with different habitats, ecology, and behavior. Variables such as migratory behavior and foraging strategies (habitat type and diet) were used because there is evidence that these behaviors are mediated by olfaction (e.g. Wenzel, 1968, 1971; Papi, 1982; Graves, 1992; Wallraff, 2003; Bonadonna et al., 2006). There is also growing evidence that olfaction is widely used in social communication and reproductive activities (see reviews Balthazart and Taziaux, 2009; Caro et al., 2015), therefore species were grouped by their mating system and social structure (group size, see below). With this suite of analyses, we demonstrate what factors have led to the diversity of OB sizes in extant birds and provide novel insight into the potential importance of olfaction in different avian lineages.

## Materials and Methods

### Specimens

Data on the size of olfactory bulbs was compiled from a total of 274 brains from 135 species of birds, which represent 21 orders. Species were grouped into orders based on Hackett et al. (2008). Specimens were obtained post-mortem from conservation authorities, wildlife veterinarians, and museum staff. Since animals were not killed to conduct this study, no university ethics approvals were required for this research. Additional data were compiled from the studies of Ebinger and Lohmer (1987), Boire (1989), Rehkamper et al. (1991), Alma and Bee De Speroni (1992), Carezzano and Bee De Speroni (1995), Fernandez et al. (1997), Pistone et al. (2002), Mehlhorn et al. (2010), Corfield et al. (2011, 2012b, 2014, 2015), Gsell (2012), Cunningham et al. (2013), and Gutierrez-Ibanez et al. (2014). Whole brain, telencephalon, and OBs sizes are included in Supplemental Table S1, which also includes the source, common names, and order for each species.

Because data is combined from multiple studies, each using different fixation protocols, it is likely that some differential shrinkage between studies has occurred. To minimize this effect, we used only the relative size of a brain structure, as all regions of a brain likely shrink by the same amount (Corfield et al., 2012a, 2014). Therefore, although the brain may have shrunk more in one study compared to another, the relative size of one structure to another within each brain will likely be comparable between studies. Coefficients of variation (CV) values, based on the ratio of log OB volume to log telencephalon volume, also suggested that within species variation was low, ranging from 0.85 to 9.70% (*n* = 6 species). For example, in mallards (*Anas platyrhynchos, n* = 4) the standard deviation was 6.5% of the mean, and in ring-necked pheasant (*Phasianus colchicus, n* = 4) and turkey (*Meleagris gallopavo, n* = 4) the value was 6.0. The CV values for these three species were calculated included measurements from Boire (1989), also suggesting that the variations between studies was low. Further, Corfield et al. (2012a) found that for most species, the variation in OB measurements among studies is low and for species represented by more than one specimen, the amount of intraspecific variation was low.

### Brain Processing

All specimens measured for this study were immersion-fixed in 4% paraformaldehyde (PFA) in 0.1 M phosphate buffer saline (PBS) for at least 1 week and then cryoprotected in a 30% sucrose solution in PBS until they sunk. Specimens were embedded in a 15% gelatin and 30% sucrose solution, placed into 4% PFA overnight, and then into 20% sucrose until the block sank. The embedded brains were sectioned on a sliding freezing microtome at a thickness of 40 μm in the sagittal or coronal plane and sections collected in PBS with 0.01% sodium azide. Every second section was mounted onto gelatinized slides, dehydrated through a graded ethanol series, cleared in Hemo-D, stained with thionin acetate (Sigma–Aldrich) and coverslipped with Permount histological mounting medium (Fisher Scientific).

### 3D Modeling

**FIGURE 1:**
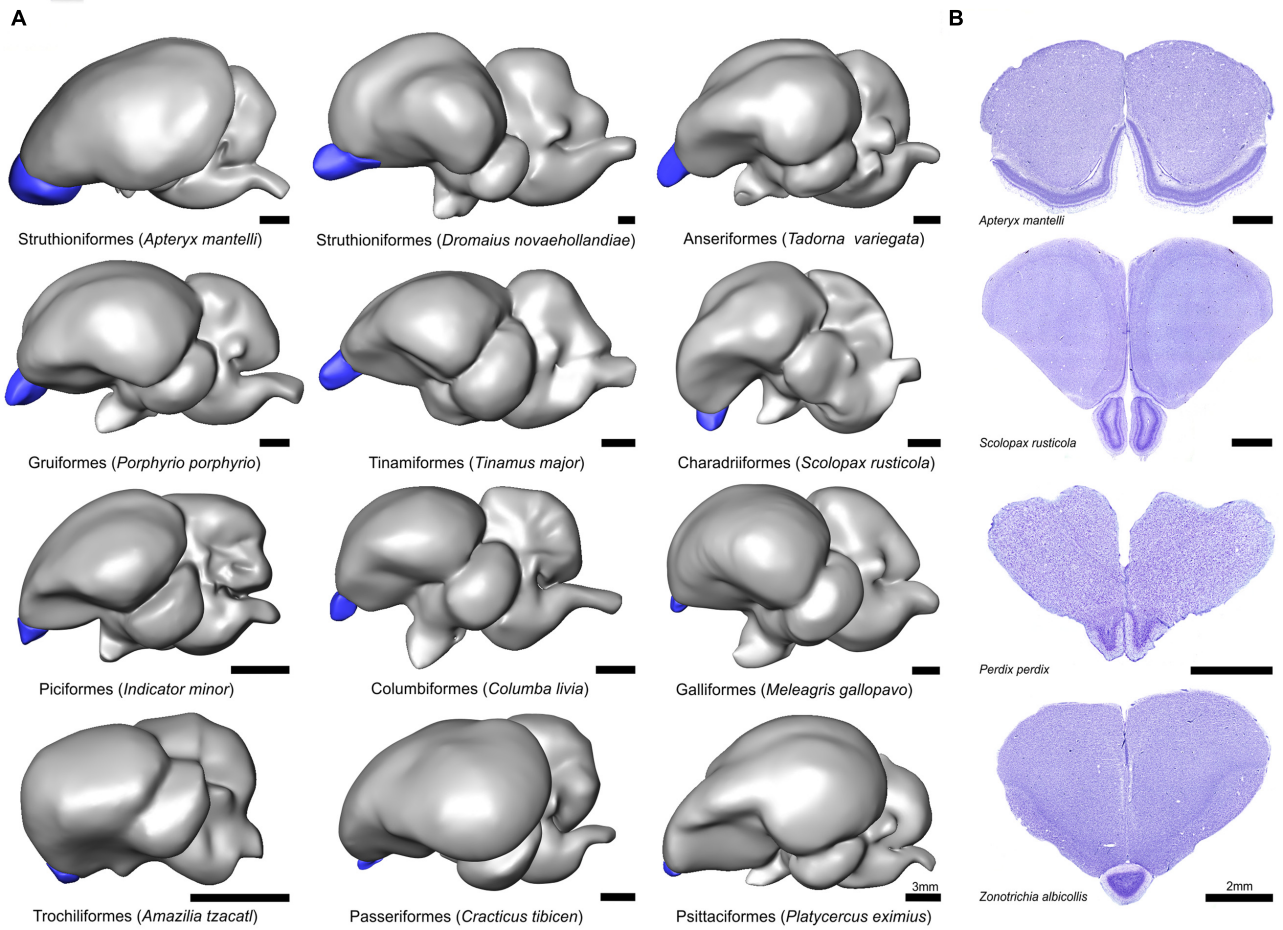
**Gross morphology of the olfactory bulbs (OBs) in birds. (A)** Lateral view of 3D models of 12 representative avian species. Blue denotes the OBs and models are organized from largest to smallest. Scale bar = 3 mm. **(B)** Coronal Nissl stained sections showing the OBs in four representative avian species and illustrating the cytoarchitectural and cross species variation. Scale bar = 2 mm.

For species shown in **Figure 1**, fiducial points were added to the gelatin block during the embedding process to align sections for 3D reconstructions (see details in Corfield et al., 2012b). In two species, *Amazilia tzacatl* and *Scolopax rusticola*, sections were aligned based on a template created from sections with fiducial points. Images of brain sections were aligned using the Alignslice module in AMIRA (v. 5.4.2, Visage Imaging, San Diego, CA, USA). To create 3D reconstructions, the LabelField module in AMIRA was used to segment out each brain region and the SurfaceGen module to create a 3D representation of the brain and OBs.

### Volumetric Measurements and Analysis

Images of Nissl sections were taken using a Retiga EXi FAST Cooled Mono 12-bit camera (Qimaging, Burnaby, BC, Canada) attached to a compound light microscope (Leica DMRE, Richmond Hill, ON, Canada) at 2.5× magnification. Brain, telencephalon, “brainrest” (which includes the brainstem, midbrain, thalamus, and cerebellum) and OB volumes were obtained directly from these images using ImageJ (1.47v, National Institutes of Health, USA). The border of the OB was defined as the inner edge of the periventricular layer and the outer edge of the olfactory fila and excluded the olfactory nerve. To obtain the volume of the OB, the cross-sectional area for each section was added to obtain a single area, which was then multiplied by the slice thickness (40 μm) and sampling interval. The number of sections containing the OB ranged from six sections (spanning 240 μm) in hummingbirds (Trochiliformes) to 44 in kiwi (spanning 1760 μm). To examine scaling relationships, we plotted the log_10_-transformed volume of the OBs against the log_10_-transformed; brain volume minus the volume of the OBs; telencephalon volume minus the volume of the OBs; and the brainrest volume.

Because phylogeny can significantly affect brain evolution (Harvey and Pagel, 1991), we first tested for phylogenetic signal using the *phytools* package in R (Revell, 2009; R Core Team, 2013) using Blomberg’s K (Blomberg et al., 2003). We found a significant phylogenetic signal for all variables (randomization test, *p* > 0.001 for all variables); therefore, we used analyses that accounted for phylogenetic effects. Phylogenetic trees were constructed based on Hackett et al. (2008) using Mesquite (v 2.75, Maddison and Maddison, 2011). Additional resolution within orders were acquired for Passeriformes (Johansson et al., 2008), Corvidae (Ericson et al., 2005), Psittaciformes (Wright et al., 2008), Anseriformes (Donne-Gousse et al., 2002), Galliformes (Wang et al., 2013), Charadriiformes (Mayr, 2011), and Tinamiformes (Bertelli and Porzecanski, 2004).

Allometric equations were calculated using phylogenetic generalized least squares (PGLS) to account for phylogenetic relatedness (Garland and Ives, 2000; Garland et al., 2005). We applied three models of evolutionary change using the *ape* (Paradis et al., 2004) and *nlme* (Pinheiro et al., 2014), packages in R: Brownian motion (BM), Pagel’s lambda (Pagel, 1999) and Ornstein–Uhlenbeck (OU) (Lavin et al., 2008; Swanson and Garland, 2009). Because the phylogeny was constructed from multiple sources, branch lengths were all set at 1, which provided adequately standardized branch lengths when checked using the procedures outlined in Garland et al. (1992). Unresolved nodes were treated as soft polytomies, with branch lengths between internal nodes set to zero (Purvis and Garland, 1993). Allometric equations were based on standard statistics, and three evolutionary models were calculated for: (1) OB volume against brain volume minus OB volume; (2) OB volume against telencephalon volume minus OB volume, and (3) OB volume against brain volume minus telencephalon (brainrest). In order to test for differences in the relative size of the OB among avian orders, we also ran regression models that included order as a covariate. Akaike Information Criterion (AIC) was used to determine which model best fit the data, with the lowest AIC considered to be the best fit (Lavin et al., 2008). Models with AIC values that differed by less than 2 units can also be considered as having substantial support (Burnham and Anderson, 2002; Duncan et al., 2007).

To examine the scaling of the OBs with brain, telencephalon, and brainrest, we also performed generalized least squares (GLS) regressions using species means in JMP v. 10 (SAS Institute). Although GLS regression does not account for the relatedness of species and treats species values as statistically independent data points, it does allow for general scaling rules across birds to be established similar to those used extensively in mammals (Herculano-Houzel et al., 2014). GLS also provides bases from which to determine how phylogenetic relationships affect the scaling of OBs.

### Ecological Categorization

Ecological data was obtained for 135 species of birds and categorized on the basis of diet, migratory behavior, habitat, mating system, social structure, and flying capabilities (Cramp, 1994; del Hoyo et al., 2014).

#### Migratory Behavior

It has long been thought that olfaction plays some role in navigation and homing in birds, whereby birds deduce positional information from airborne odors carried by winds to find their way home (e.g., Papi, 1976, 1982; Wallraff, 2004, 2013). Navigation by olfactory cues has been shown in many species ranging from swifts, *Apus apus* (Fiaschi et al., 1974), starlings, *Sturnus vulgaris* (Wallraff et al., 1995), catbirds *Dumetella carolinensis* (Holland et al., 2009), pigeons *Columba livia* (Papi, 1976; Wiltschko and Wiltschko, 1992; Benvenuti and Ranvaud, 2004) and many seabirds (Gagliardo et al., 2013). However, there is some skepticism with respect to olfactory mediated navigation (e.g., Gould, 2009; Jorge et al., 2009, 2010; Wiltschko, 2012; Blaser et al., 2013; Phillips and Jorge, 2014; Wallraff, 2014), therefore determining if migration behavior correlates with relatively large OBs might shed some light on this argument. Larger OBs might give migratory species an improved ability to decode and map patterns of odorants, allowing for example, locations to be mapped to olfactory space, an aspect that is crucial for both short and long-distance navigation (Jacobs, 2012). Migratory behavior was grouped into three categories; (1) those that have regular seasonal migration, normally traveling 1000s of kilometers between breeding and wintering grounds. (2) those that have more localized movement within an area, normally driven by seasonal changes in food supply. (3) those that are sedentary, residing in a single location and normally maintaining a year round territory.

#### Social Communication and Reproductive Behaviors

Studies demonstrating the role of olfaction in sociality and reproduction in birds are growing and it is clear that in some species it plays an important role (see review, Balthazart and Taziaux, 2009; Caro and Balthazart, 2010; Caro et al., 2015). For example; mate recognition has been demonstrated in Antarctic prions (*Pachyptila desolata*, Bonadonna and Nevitt, 2004), olfactory cues have been linked to reproductive behaviors in mallards (*Anas platyrhynchos*, Balthazart and Schoffeniels, 1979; Caro and Balthazart, 2010) and the distinctive tangerine odor of crested auklets *(Aethia cristatella)* has been suggested to play a social and reproductive role (Hagelin et al., 2003). As categories for social communication and reproductive behaviors we used: (1) social mating system, where birds were categorized as monogamous (one mate during their lifetime) or polygamous (more than two mates), and (2) social complexity, where birds were organized by group size, which is often used as a measure of social complexity (Dunbar, 1995; Burish et al., 2004; Lehmann and Dunbar, 2009). For social complexity, we categorized birds as: solitary (1 to 3 birds), covey (5 to 50 birds) or colonial (100s to 1000s of birds) following the scheme of Burish et al. (2004). However, unlike Burish et al. (2004), we including breeding-ground gatherings in our assessment of group size; for example if a species is solitary for most of its life, but comes together to breed in groups of ∼1000, this species would be categorized as colonial. In species that gather in large groups or colonies, such as most shorebirds and seabirds, chemosignals could be utilized for many behaviors including territoriality, attraction, individual, species, and kin recognition, hierarchical status, and mate-choice (see reviews Balthazart and Taziaux, 2009; Caro and Balthazart, 2010; Caro et al., 2015), therefore enhanced olfactory capabilities might be expected in these species.

#### Foraging Strategies

Olfaction is widely accepted to play a significant role in foraging and has been reported in a number of birds, including kiwi (*Apteryx australis*, Wenzel, 1968, 1971), and vultures (Stager, 1964; Graves, 1992), and is also well documented in seabirds (Grubb, 1972; Hutchison and Wenzel, 1980; Lequette et al., 1989; Verheyden and Jouventin, 1994). We split foraging strategies into two categories; diet and habitat type. For diet, species were categorized into those that are carnivores, herbivores, insectivores, and piscivores. Habitat type was broadly defined as either species that were (1) semi-aquatic, meaning they utilized the ocean, rivers, lakes, or wetlands for foraging, (2) aquatic, meaning they spent most of their life in water, such as penguins, and (3) terrestrial, meaning that they live and forage almost exclusively on land.

#### Flying Capabilities

In a recent study done by Corfield et al. (2014) some of the largest relative OB sizes were found in kiwi and emu (*Dromaius novaehollandiae*), both flightless and also two gruiform species (*Fulica armillata* and *Porphyrio porphyrio melanotus*), which have poor flying abilities. This putative correlation may suggest that enhanced olfactory capabilities have evolved in species that spend their entire life on or near the ground, although this has yet to be tested on a large scale. We therefore categorized species by their flying capabilities; (1) those that are flightless, having wings that are incapable of flight, (2) those with poor flight, spending most of the time on the ground, but are capable of short bursts of flight if disturbed or to reach roosts, (3) those that are aerial and are capable of maintained flight.

To examine whether relative OB size varied significantly with the behavioral categories, we used PGLS models with the categories as covariates. For simplicity, we only show Pagel’s (1999) transformation as the evolutionary model, but the results were qualitatively the same when we applied other evolutionary models. As before, AIC was used to determine which model best fit the data. Additionally, we used likelihood ratio tests to compare the fit of each model against the null model (OB vs. brain volume).

#### Visual and Tactile Brain Regions

It is clear from studies in fish and mammals that brain regions compete with one another for their proportion of total brain mass (Jerison, 1973; Yopak et al., 2010). Although this has yet to be tested in birds, it is possible that variation in OB sizes could be as a result of the relative size of other sensory systems. For example, owls have a massive thalamofugal visual pathway and large auditory nuclei, which may have resulted in their relativity small OBs, optic tectum (TeO) and principal sensory nucleus of the trigeminal nerve (PrV) (Kubke et al., 2004; Iwaniuk et al., 2006, 2008; Martin et al., 2007; Gutierrez-Ibanez et al., 2009). Therefore, we measured the volumes of the PrV, and TeO, which receive projections from the beak (Dubbeldam, 1990) and retina (Hunt and Webster, 1975), respectively, to determine whether relative OB sizes are correlated with the relative size of either of these brain regions. Measurements were obtained from the same specimens that OB volumes were obtained and using the same method as outline above. We also included data from Gutierrez-Ibanez et al. (2009, 2014) and Cunningham et al. (2013), giving us data from 91 species in total. Phylogenetically corrected residual analysis was carried out for both PrV and TeO as above and species with positive residuals were categorized as having a large brain region and those with negative as small.

### Trait Mapping

We use the *contmap* function of the *phytools* R package (Revell, 2012) to visualize changes in the relative size of OBs through phylogeny. The *contmap* function maps a continuous trait, in this case the relative size of OB, onto the phylogeny by estimating the ancestral states at the internal nodes using maximum likelihood and interpolating the states along each edge using Eq. 2 of Felsenstein (1985; Revell, 2013). The relative size of OBs are expressed as the residuals of the best fitting PGLS model. Although we created plots for all three scaling variables, they were all nearly identical and species values were all strongly correlated with one another. Therefore only the plot for brain volume is shown.

We also took the order averages of the relative size (expressed as the residuals of the best fitting PGLS model of each area against brain volume) of each sensory area (OB, PrV, and TeO) and used the *fancyTree* function of *phytools* (Revell, 2012) to map each trait onto the phylogeny, as above. We also used this package to plot one trait against the other, while mapping the phylogenetic relationship of orders. Phylogenetic relationships are projected into the Cartesian space and are indicated by line connections. For example, OB was plotted against the TeO, with lines collecting closely related species, such as galliforms and Anseriformes.

## Results

### OB Morphology

There was considerable morphological variation in the relative size and shape of OBs within and across orders (**Figure 1**). Perhaps the most divergent shape of the OBs is that of kiwi; the OBs extend over the majority of the rostral telencephalon and form an extensive cortex-like sheet (**Figure 1**, Corfield et al., 2014). A stalk-like or ‘pedunculated’ structure is typical of all other birds examined, although the shape and anatomical location differs among species, together with obvious relative size differences (see below). The telencephalon of the Eurasian woodcock (*Scolopax rusticola*), for example, is pitched forward, likely due to their eyes being set far back in their head (Martin, 1994), resulting in a more ventrally placed OB. In all 33 songbird species examined, including corvids, the OBs are fused. An example of the OBs of the white-throated sparrow (*Zonotrichia albicollis*) is shown in **Figure 1**. The laminar organization of OBs was highly conserved across the species examined in this study, including kiwi (**Figure 1B**). The external plexiform, mitral cell, internal plexiform, granule cell, and periventricular layers were all visible in Nissl sections, although there were obvious differences in regards to the relative thickness and cell density of layers.

#### Scaling of the OB with Brain Size

**FIGURE 2:**
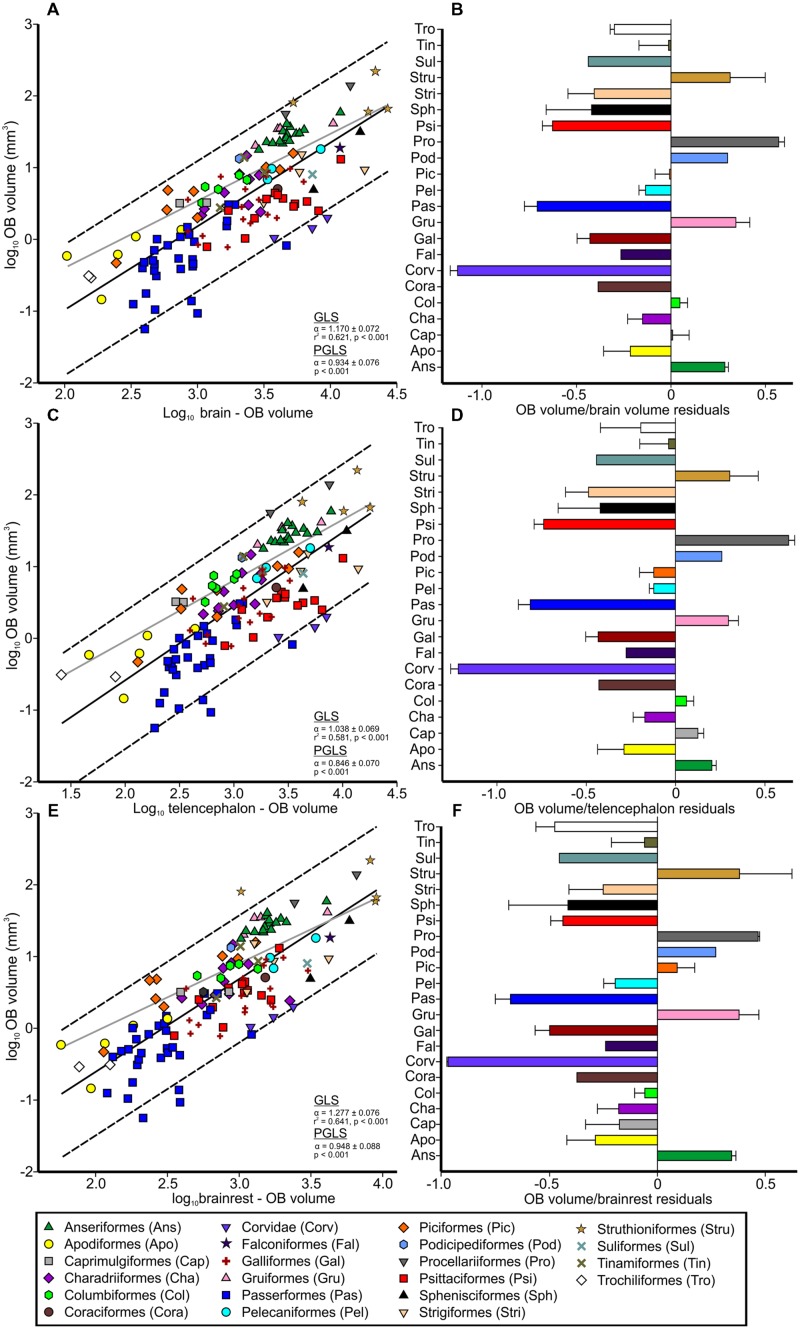
**Relative size of the OBs in birds.** Scatterplots of the log-transformed volume of the OBs plotted as a function of either: **(A)** the log-transformed brain volume minus the volume of the OBs, **(C)** the log-transformed telencephalon volume minus the volume of the OBs or **(E)**; the brainrest (brainstem, cerebellum, and thalamus) volume. The solid lines indicate the results from the generalized least-squares (GLS) regression, the broken lines the 95% prediction intervals and the gray lines the results from the phylogenetic generalized least squares (PGLS) model. Symbols denote order as presented in legend. Bar graphs show the size of OBs relative to: **(B)**; the brain, **(D)**; the telencephalon; and **(F)** the brainrest, with bars representing the order means (with SD) of the residuals derived from the respective regressions.

Brain volumes ranged from 103.68 mm^3^ in pygmy swiftlets (*Collocalia troglodytes*) to 27006.26 mm^3^ in ostriches (*Struthio camelus*), constituting a 260-fold increase, whereas the volume of the OBs varied from 0.06 mm^3^ in spotted pardalotes (*Pardalotus punctatus*) to 217.63 mm^3^ in emus, constituting a 3627-fold increase in size. When accounting for phylogeny, the volume of the OBs significantly varied as a function of brain, telencephalon, and brainrest volumes (*p*’s < 0.001, *F*’s = 3.98–39.00, **Figures 2A,C,E**). The OBs scaled with the telencephalon with negative allometry (α = 0.846 ± 0.070, **Figure 2C**), suggesting that the OBs gain volume slower than the telencephalon does. The exponent was closer to isometry for the brain (α = 0.934 ± 0.076, **Figure 2A**) and brainrest (α = 0.948 ± 0.088, **Figure 2E**), indicative of a linear relationship where the OBs gain mass at the same rate as does the brainrest and brain as a whole. For GLS regressions, the volume of the OBs also significantly varied as a function of brain, telencephalon and brainrest volumes (*p*’s < 0.001, *F*’s = 184.940–237.581, df = 1,134), and scaled with positive allometry (**Figures 2A,C,E**). The OBs scaled with brain volume (α = 1.170 ± 0.072, CI = 1.011–1.324, **Figure 2A**) and brainrest (α = 1.277 ± 0.076, CI = 1.126–1.458, **Figure 2E**) with positive allometry, suggesting that the OBs gain volume faster than does the brainrest and brain as a whole. The OBs scaled with the telencephalon close to isometry (α = 1.038 ± 0.069, CI = 0.883–1.183, **Figure 2C**). Compared to a GLS approach, PGLS suggests that the OBs scaled with all three variables at a slower rate, with the telencephalon scaling at the slowest rate. This suggests that phylogenetic relationships among birds are effecting how the OBs scale with brain size.

The *r*^2^ values for the OBs against brain, telencephalon, and brainrest were 0.621, 0.581, and 0.64, respectively, suggesting that some of variation in the volume of OBs cannot be explained by brain, telencephalon, or brainrest volume.

#### Scaling of the OB Within Orders

Scaling of the OBs was examined individually for ducks (Anseriformes), shorebirds (Charadriiformes), pigeons (Columbiformes), galliforms, songbirds (Passeriformes, with and without corvids and *Cracticus tibicen*), honey guides, woodpeckers, barbets, and toucan (Piciformes), parrots (Psittaciformes), and ratites + tinamous (Struthioniformes + Tinamiformes) (**Table 1**). The volume of the OBs varied significantly as a function of the brain volume in all orders (*p* < 0.05) except pigeons (*p* = 0.094, *F* = 4.776, df = 1,5) and shorebirds (*p* = 0.164, *F* = 2.350, df = 1,9, *r*^2^ = 0.227). In shorebirds, little of the variation in OB volumes could be explained by brain volume (*r*^2^ = 0.227), whereas in honey guides, woodpeckers, barbets, and toucan, most of the variation could be explained by brain volume (*r*^2^ = 0.806).

**Table 1:** Scaling of the olfactory bulbs (OBs) with brain, telencephalon, and brainrest for nine avian orders.

	Ans	Cha	Col	Gal	Pas-Corv	Pas	Pic	Psi	Stur
**Brain volume**					
*r*^2^	0.720	0.230	0.540	0.420	0.381	0.338	0.810	0.590	0.730
Slope	0.790	0.850	0.800	0.860	1.261	0.680	0.920	0.830	1.090
SE	0.135	0.553	0.368	0.271	0.315	0.174	0.185	0.207	0.294
*F*	34.000	2.350	4.770	10.170	15.989	15.317	24.930	16.070	13.770
*p*	<0.001	0.160	0.090	0.007	<0.001	<0.001	0.003	0.002	0.010
**Telencephalon volume**					
*r*^2^	0.592	0.494	0.534	0.399	0.427	0.356	0.791	0.588	0.789
Slope	0.615	1.085	0.901	0.802	1.254	0.646	0.808	0.757	1.087
SE	0.142	0.389	0.421	0.263	0.286	0.159	0.170	0.191	0.251
*F*	18.852	7.805	4.584	9.281	19.355	16.559	22.646	15.681	18.700
*P*	<0.001	0.023	0.099	0.009	<0.001	<0.001	0.003	0.002	0.008
**Brainrest**					
*r*^2^	0.684	0.027	0.471	0.433	0.290	0.311	0.813	0.540	0.546
Slope	0.787	0.234	0.632	0.910	1.161	0.755	1.208	1.055	0.951
SE	0.148	0.496	0.334	0.278	0.356	0.205	0.236	0.294	0.388
*F*	28.089	0.223	3.568	10.682	10.615	13.519	26.099	12.901	6.001
*p*	0.000	0.649	0.132	0.006	0.003	0.001	0.002	0.004	0.058
df	1, 14	1, 9	1, 5	1, 15	1, 27	1, 31	1, 7	1, 12	1, 6

*Data is shown for Anseriformes (Ans), Charadriiformes (Cha), Columbiformes (Col), Galliformes (Gal), Passeriformes, with (Pas) and without corvids and *Cracticus tibicen* (Pas-Corv), Piciformes (Pic), Psittaciformes (Psi) and Struthioniformes + Tinamiformes (Stur).*

**FIGURE 3:**
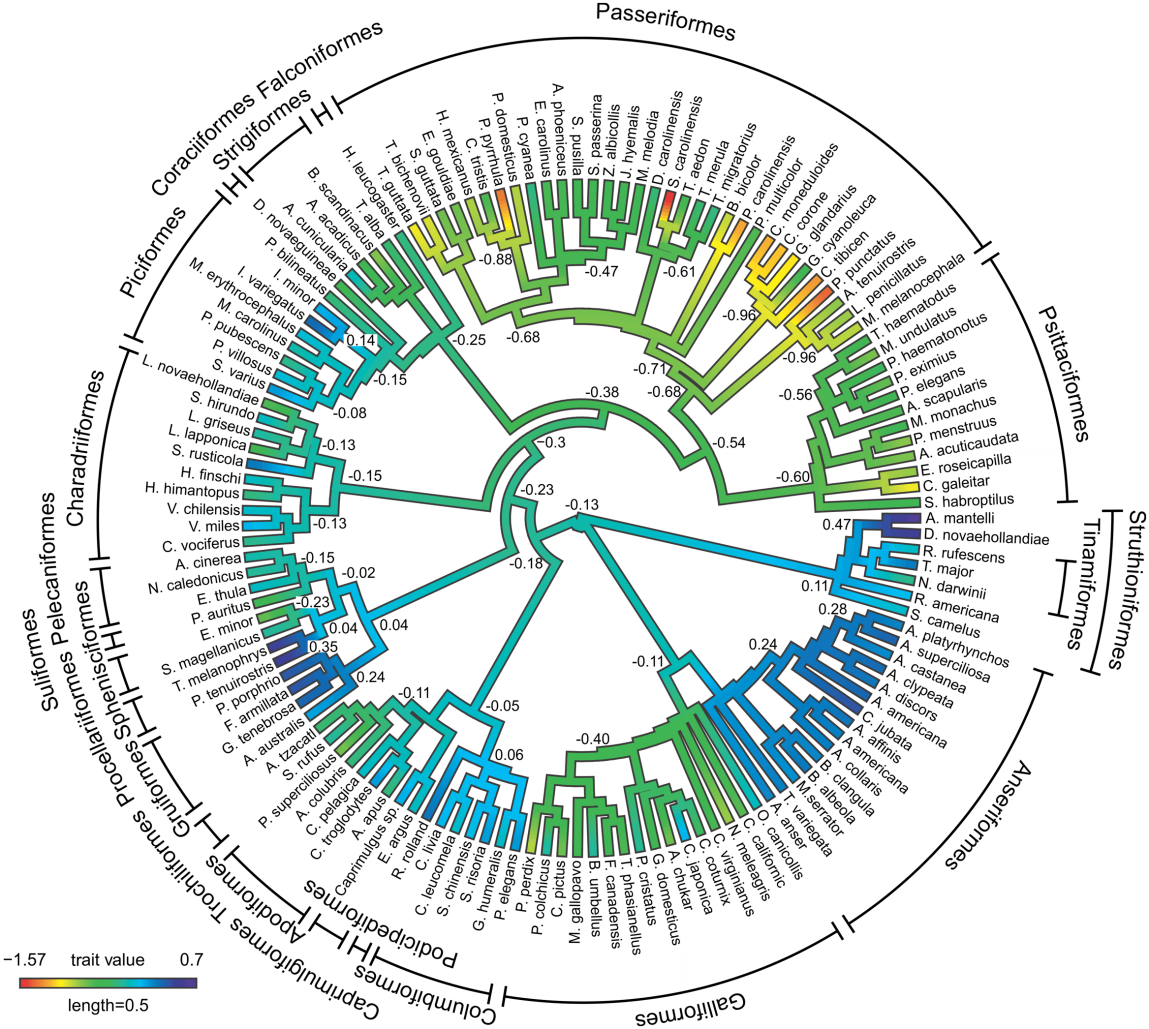
**Ancestral character estimates for OB volumes plotted onto an avian phylogeny.** This method uses ancestral character estimation to visualize historical character states for OB volumes (plotted as a continuous trait) along the branches of a tree (see methods, Revell, 2013). The trait mapped was the relative size of OBs expressed as the residuals of the best fitting PGLS model of OBs against brain volume-OBs. The phylogeny is largely based on Hackett et al. (2008), with further resolution coming from other studies (see Materials and Methods).

In most orders, the OBs scaled with brain, telencephalon, and brainrest volume isometrically, more so than the relationship across all species (**Table 1**). There were, however, some exceptions. In ducks, the OBs scale with negative allometry (Brain; α = 0.790 ± 0.135, Tel; α = 0.615 ± 0.142, Brainrest; α = 0.787 ± 0.148), suggesting that the OBs gain volume slower than the brain (especially the telencephalon) compared to that of other species. When all songbirds are included in the regression analysis, the OBs scale with brain, telencephalon and brainrest volume with negative allometry (Brain; α = 0.680 ± 0.174, Tel; α = 0.646 ± 0.159, Brainrest; α = 0.755 ± 0.205, **Figure 3**, **Table 1**). However, when corvids and the Australian magpie (*Cracticus tibicen*) are removed from the analysis, the exponents shift to an isometric relationship (Brain; α = 1.261 ± 0.315, Tel; α = 1.254 ± 0.286, Brainrest; α = 1.161 ± 0.356).

#### Species and Order Variations in OB Size

Nearly all bird species fall inside of the 95% prediction intervals, suggesting that the OBs scale with brain size in a reasonably predictable manner across birds (**Figure 2**). The only exceptions to this were kiwi, with hypertrophied OBs when regressed against brainrest, and some songbirds, which have hypotrophied OBs. Indeed, the residuals for the songbirds were the smallest of all species examined in this study (**Figures 2B,D,F**). Parrots, penguins, owls, and galliforms were also characterized by small OBs whereas ducks, seabirds, rails and ratites by large OBs (**Figure 2**).

To further examine how phylogeny relates to variations in OB sizes, we plotted the residuals of OB vs. brain (as a continuous character) onto a phylogeny and determined likely ancestral states (**Figure 3**). In all ratites, the OBs have increased in size from the predicted ancestral condition, with a further increase in the ancestor of kiwi and emu (0.47). The OBs in the three species of tinamou varied from large in the great tinamou (*Tinamous major*, 0.28), to small in Darwin’s Nothura (*Nothura darwinii*, –0.22). The OBs of all duck species have increased in size (0.19–0.45), however, OBs in the closely related galliforms are small and are reduced in size (–0.86–0.09) compared to the predicted ancestral condition. Rails and seabirds are characterized by some of the largest OBs found in this study (0.37–0.60), with pigeons, honey guides (*Indicator* sp., especially *I. variegatus*), Eurasian woodcock (*Scolopax rusticola*) and white-tufted grebe (*Rollandia rolland*) all characterized by moderately large OBs. Songbirds and parrots were clearly characterized by small OBs, with many instances where specific species have undergone an even further reduction in OB sizes. For example; Eurasian bullfinch (*Pyrrhula pyrrhula*), white-breasted nuthatch (*Sitta carolinensis*), Carolina chickadee (*Poecile carolinensis*) and spotted pardalote have some of the smallest relative OB sizes found in this study (–1.35, –1.57, –1.22, and –1.41, respectively). It appears that the common ancestor of corvids (*Corvus* sp. and *Garrulus glandarius*), Australian magpies and magpie-larks (*Grallina cyanoleuca*) had small OBs (–0.96), with the slightly larger OBs of magpie-larks (–0.48) likely a derived trait.

#### Variation in OB Size in Relation to Ecology

**FIGURE 4:**
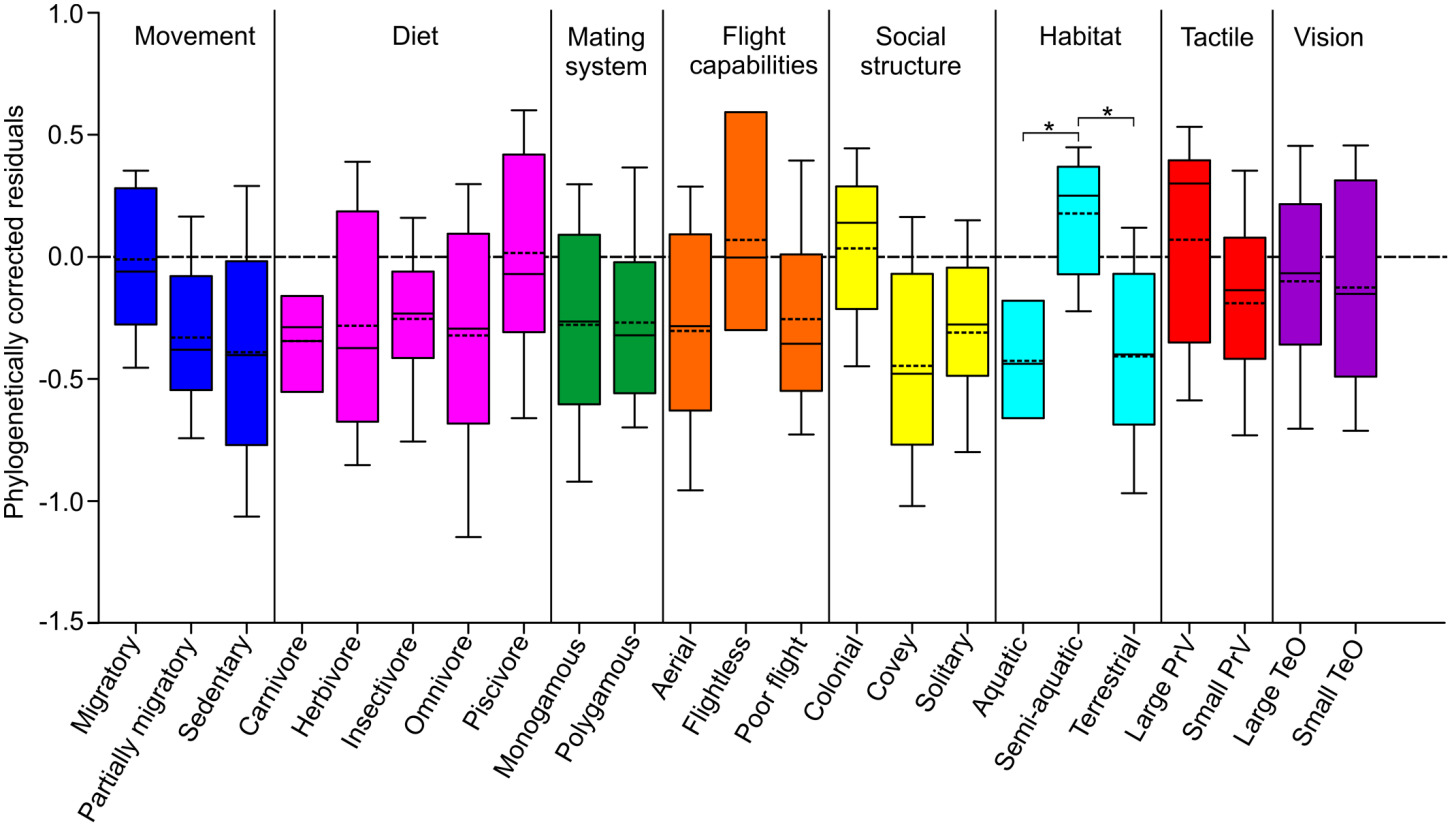
**Variation in OB sizes in relation to ecology and behavior.** Box and whisker plots show the variation in OB volumes among the different ecology and behavior categories, as calculated from phylogenetically corrected residuals. For each ecological niche category, the ‘box’ represents the second quartile and the error bars (‘whiskers’) are the first and third quartiles. The horizontal bar in each box represents the median, while the broken line indicates the mean. Asterisks indicate statistically significant differences (*p* < 0.05).

Relative OB sizes were analyzed, while taking into consideration phylogenetic relationships, to determine if variations in size could be correlated with specific ecological variables (**Figure 4**). The OB residuals for species that inhabited a semi-aquatic (median 0.25, mean 0.18) were mostly positive whereas the residuals in aquatic (median –0.44, mean –0.43) and terrestrial (median –0.41, mean –0.40) species were mostly negative. A statistically significant effect was found for habitat (*p* = 0.008, λ = 0.922), suggesting that semi-aquatic species have larger OBs than both aquatic and terrestrial species. The OB residuals for species with a large PrV (median 0.30, mean 0.07) were also mostly positive and larger than species with a small PrV (median –0.14, mean –0.19), however, there was no statistically significant effect found for PrV size (*p* = 0.087, λ = 0.687). Migratory (median –0.06, mean –0.01) and colonial (median 0.14, mean 0.03) species and those that are flightless (median 0.00, mean 0.07) and piscivorous (median –0.07, mean 0.02) also appeared to have larger OBs than the other species in their respective categories, however, there was also no statistically significant effect found for any of these categorizes (movement; *p* = 0.513, λ = 0.887, social structure; *p* = 0.253, λ = 0.890, flight capabilities; *p* = 0.359, λ = 0.917, diet; *p* = 0.308, λ = 0.926).

**FIGURE 5:**
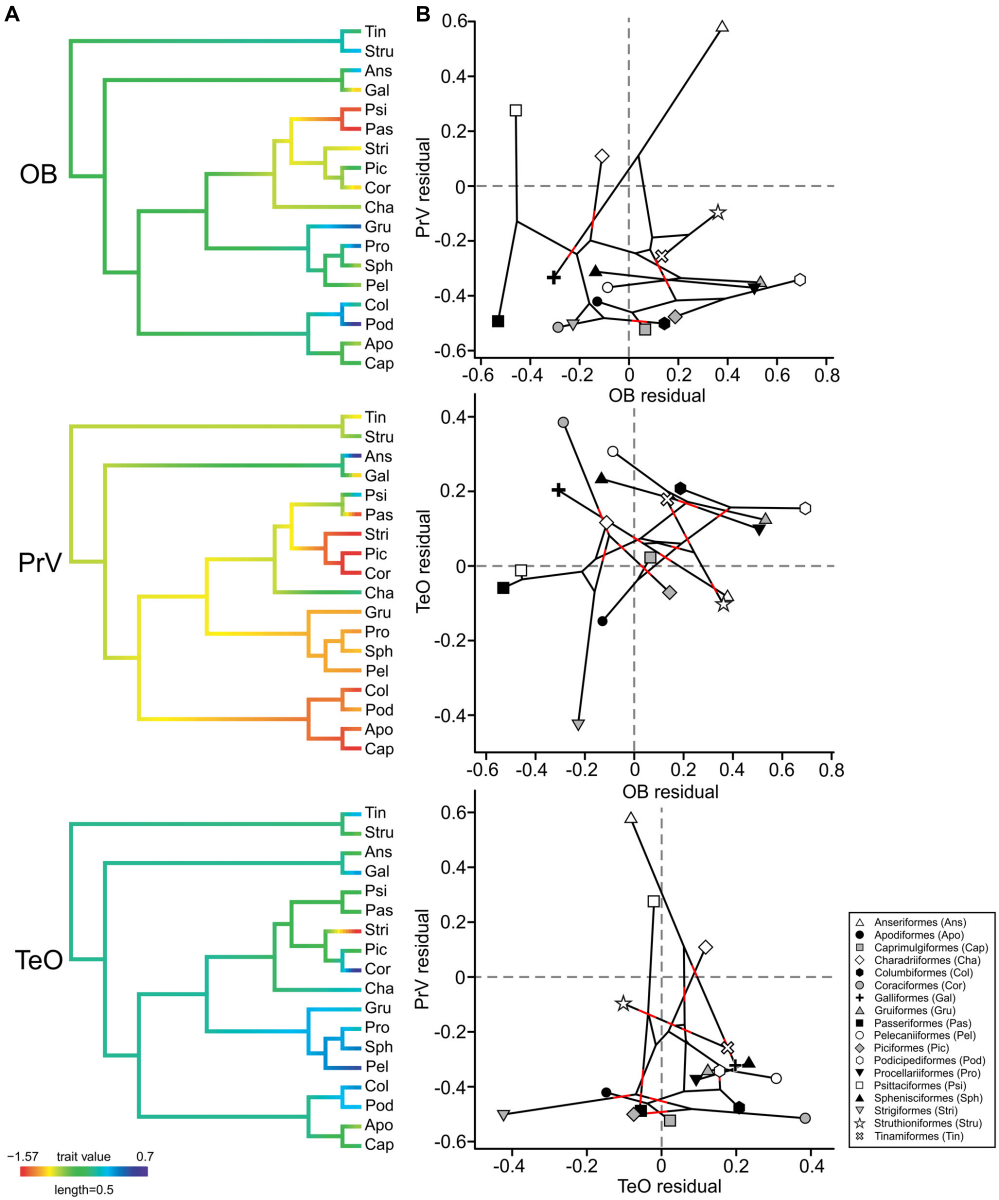
**Ancestral character estimates and residual plots for the OBs, the principal sensory nucleus of the trigeminal nerve (PrV), and the optic tectum (TeO). (A)** Ancestral character estimates are plotted using the order averages for the relative size (expressed as the residuals of the best fitting PGLS model of each area against brain volume) of each sensory area (OB, PrV, and TeO) and are mapped onto the phylogeny based on Hackett et al. (2008). **(B)** Plots showing each sensory area plotting against the other while indicating the phylogenetic relationship of species (black lines). Red lines have been added to areas where the lines cross to allow for the tree branches to be more easily identified.

To explore whether the size of other brain regions that process sensory information may be contributing to variations in the size of OBs, we used PGLS to test for correlations between the residuals of OBs and the PrV and TeO. There is a significant and negative correlation between the size of TeO and PrV (α = –0.217 ± 0.068, *p* = 0.002), suggesting that as one brain region increases in size the other decreases in size. No other significant correlations were found (OB/PrV, α = 0.203 ± 0.115, *p* = 0.079 or OB/TeO, α = –0.067 ± 0.062, *p* = 0.281), although there is some evidence to suggest a positive correlation between OB and PrV size, mostly driven by ducks, which have a large PrV and also large OBs (**Figure 5**). Ducks had both larger PrV and OB sizes compared to the closely related galliforms, whereas in galliforms the TeO was among the largest of any order. Parrots and songbirds had similar small OB sizes and an average TeO, whereas parrots had a large PrV and songbirds a small PrV. Seabirds, rails and grebes all had large OBs and TeO, whereas their PrV was small. No orders had obvious enlargements to all three brain regions.

## Discussion

Overall, the size of avian OBs varies predominately with the size of the brain. This allometric relationship was for the most part isometric; the OBs gain volume at the same rate as the rest of the brain does, although there was some indication that the OBs gain volume faster than does the brain as a whole. A similar relationship has also been described in fish and some mammals, although there is a tendency for a negative allometic relationship; OBs gain mass slower than the rest of the brain (Gittleman, 1991; Yopak et al., 2015). Another feature of the OBs in mammals and fish is that they do not scale as tightly with brain size as other brain regions do and have a substantial level of allometric independence from the rest of the brain (Finlay and Darlington, 1995; Finlay et al., 2001; Reep et al., 2007; Gonzalez-Voyer et al., 2009; Yopak et al., 2010, 2015). In birds too, not all of the variation in OB sizes can be explained by brain size alone with *r*^2^ values that were between 0.58 and 0.64 and the data widely scattered. Interestingly, *r*^2^ values for some mammals (Carnivora = 0.80, primates = 0.56, bats = 0.85, insectivores = 0.83, Gittleman, 1991; Barton et al., 1995) and fish (GLS = 0.73, PGLS = 0.69, Yopak et al., 2015) are generally higher than we show in birds, indicating that in birds the size of OBs is less dependent on the size of the rest of the brain than it is in both mammals and fish. We suggest that much of this variation can be attributed to species differences in phylogeny, habitat, sensory ecology, and behavior, as is the case in mammals and fish (see below).

It should be noted that the study of Zelenitsky et al. (2011), which analyzed the OB ratios of Bang and Cobb (1968), showed that in birds the OBs are not correlated with body size and virtually none of the variation in OB sizes is explained by body size (*p* = 0.26, *r*^2^ = 0.009). Why a significant correlation was not found in Zelenitsky et al. (2011) could reflect the fact that body size rather than brain size was used to examine allometric scaling, and also due to the methodological limitations of the Bang and Cobb data set (see Corfield et al., 2014; Caro et al., 2015). A study comparing the Bang and Cobb data set and the volumetric measurements obtained in the current study is indeed warranted to determine the validity of their methods.

Phylogenetic relationships and evolutionary history have undoubtedly been factors influencing the size of OBs in extant birds (see **Figure 3**). It is apparent that OBs in more basal species, including ratites, ducks, pigeons, rails, and seabirds are generally large, whereas OBs have been reduced in more diverged taxa, namely songbirds and parrots. Large OBs in these basal species have been inherited from early neornithines, where improved olfaction evolved for more effective foraging and/or navigation skills (**Figure 3**, Bang and Cobb, 1968; Wenzel, 1971; Zelenitsky et al., 2011). A shift away from olfaction has then occurred in more derived taxa during the evolution of visual and vestibular sensory enhancements association with flight (Wenzel, 1971; Alonso et al., 2004; Milner and Walsh, 2009). Probably the most interesting feature in the evolution of OB sizes across birds is in cases where sister taxa have diverged in OB sizes, namely between ducks and galliforms, penguins and seabirds, and honey guides and woodpeckers. In these cases it is clear that there have been opposing factors either driving the evolution of large or small OBs. In seabirds and honey guides, foraging based on olfactory cues has likely led to large OBs compared to their sister groups, which forage using visual and tactile cues (see below, Isack and Reyer, 1989; Nevitt, 1999; Short and Horne, 2001; del Hoyo et al., 2014). What evolutionary mechanisms have led to small OBs in galliforms is not obvious, however, in ducks large OBs may have evolved to utilize olfactory cues in a semi-aquatic environment (see below). In addition to differences between taxa, there are also many instances where a single species within a taxa stands out as having either small or large OBs, such as the small OBs in spotted pardalotes and the large OBs in Eurasian woodcocks. Such diversification in OBs size both among and within taxa suggests that olfaction is highly adaptable, with increases and decreases in olfactory capabilities evolving to meet the specific sensory demands of an ecological niche or a certain behavior.

Of all of the ecological/behavioral variables that we tested, only habitat was significant; semi-aquatic species had significantly larger OBs than terrestrial or aquatic species. Although a semi-aquatic habitat is a broad definition and indeed encompasses habitats ranging from rivers, lakes, oceans and wetlands, these habitats may provide olfactory cues that are particularly important for the birds that occupy them. Petrels, albatross, and prions, for example, can detect and localize high prey abundance using the odors (dimethyl sulfide and pyrazines) produced by phytoplankton when grazed by zooplankton as they forage out at sea (e.g., Grubb, 1972; Hutchison and Wenzel, 1980; Nevitt et al., 1995; Nevitt, 2000; Nevitt and Haberman, 2003; Nevitt and Bonadonna, 2005; Bonadonna et al., 2006; Dell’Ariccia and Bonadonna, 2013). The same likely applies to other semi-aquatic and also aquatic species, however, to date this has only been shown in penguins (Spheniscidae), which use dimethyl sulfide as a behavioral cue to track upwind plankton blooms over long distances (Culik, 2001; Cunningham et al., 2008; Wright et al., 2011).

Interestingly, large relative OBs are not generally found in semi-aquatic mammals and instead have smaller relative OBs compared to terrestrial mammals (see review Pihlström, 2008). Relatively small OBs are found in otters (Mustelidae, Radinsky, 1968; Gittleman, 1991), aquatic insectivores (Sánchez-Villagra and Asher, 2002), pinnipeds (Repenning, 1976; Reep et al., 2007) and also platypus (*Ornithorhynchus anatinus*) (Pirlot and Nelson, 1978). Because early tetrapods evolved a new set of olfactory receptor molecules that were adapted to detecting airborne odors, mammals that moved back to an aquatic environment lacked the ability to detect scents underwater (Freitag et al., 1995, 1998). Therefore in mammals, the use of olfaction in semi-aquatic and aquatic habitats is limited and indeed in some aquatic mammals, such as toothed whales (Odontocetes), OBs and nerves are essentially absent or greatly reduced (Edinger, 1955; Breathnach, 1960; Morgane and Jacobs, 1972; Pirlot and Kamiya, 1985; Cave, 1988; Ridgway, 1990; Oelschlager and Kemp, 1998; Manger, 2006). Birds too are constrained by an ancestor that was adapted to detecting airborne odors, and indeed species that are aquatic and forage exclusively underwater, such as penguins and cormorants, have some of the smallest relative OBs, second only to songbirds (**Figure 2**). In these species, the opening of the external nares are reduced, functionally closed or an epithelial nasal valve is present. Although this is a necessary adaptation for diving underwater, it greatly reduces airflow to the caudal nasal concha (Bang, 1971; Bang and Wenzel, 1985). An exception to this is, however, found in shearwater and diving ducks, which do not have reduced OBs, yet forage almost exclusively by diving.

Given that semi-aquatic mammals and both aquatic mammals and birds have poor olfactory capabilities, why then do many semi-aquatic birds have a good sense of smell? Certain aspects of the behavioral ecology of birds, such as flight, have undoubtedly allowed some birds to evolve chemosensitive adaptations to take advantage of the olfactory cues in a semi-aquatic environment. Indeed, in some semi-aquatic species an enlargement to the olfactory system most certainly is a functional adaptation associated with this habitat, providing cues for navigation (Jacobs, 2012) and foraging in an otherwise featureless environment. The link between large relative OBs and a semi-aquatic environment is hard to pinpoint in other species, including ducks, rails and shorebirds. Indeed, based on the foraging behavior in these species, and also the patterns observed in mammals, it would be predicted that they would have small OBs. One possibility is that large OBs have not been driven solely by occupying a semi-aquatic habitat, but are also due to a combination of other behaviors. For example in ducks, olfaction is utilized to determine endocrine condition (Balthazart and Schoffeniels, 1979; Jacob et al., 1979; Balthazart and Taziaux, 2009; Caro and Balthazart, 2010), could be used for determining hierarchical status when in colonials (Balthazart and Taziaux, 2009; Caro and Balthazart, 2010; Caro et al., 2015) or could be used to decode and map patterns of odorants for long range migration (Jacobs, 2012).

In addition to neural scaling, phylogeny and ecology/behavior, competition for brain space is another factor that is possibly causing variations in OB sizes among birds. Strong evidence of an apparent tradeoff, where the size of the OBs is negativity or positively correlated with the size of the PrV or TeO was not found in birds. However, there were instances of small OBs in species with a large TeO and/or PrV. For example, parrots have one of the largest PrV sizes of any bird and also some of the smallest OBs, possibly indicating that increased beak sensitivities (Gutierrez-Ibanez et al., 2009; Demery et al., 2011) have led to reduced olfactory capabilities. As noted by Zelenitsky et al. (2011), the small OBs in parrots and also corvids could have also resulted from the extra neural demands associated with their increased cognitive abilities (Emery, 2006). We do, however, show a significant correlation between the size of PrV and TeO, suggesting that species with large PrV have small TeO and vise versa. This was particularly true in shorebirds, where this neural trade off can be seen in difference in feeding ecology; some species rely mostly on vision whereas others locate food with a tactile organ in their beak tips (Piersma, 2011; Cunningham et al., 2013). Therefore in birds, it is likely that neural structures do compete for brain space, although OBs do appear to be more free to vary in size irrespective of the size of other sensory structures, which is also true in fish (Yopak et al., 2010, 2015).

Overall, in birds it is clear that many factors have led to the diversity in OB sizes in birds. On the one hand, overall brain size is an important factor in shaping the size of the OBs and so too is an evolutionary history that points to an ancestor with large OBs. On the other hand, habitat and behaviors such as migration, foraging strategies, and social structure are playing a role in driving enhancements to the olfactory system. In addition, the relative importance of other sensory regions needs to be considered as there is competition for a limited amount of brain space. It can therefore be concluded that multiple factors have in some way contributed to the diversity in OB sizes seen across birds and that it is important to consider all of these variables before we can fully understand the mechanisms driving the evolution of olfaction. By examining the OB sizes across such a diverse array of avian species we have helped to confirm that olfaction is indeed a functional sense in birds.

## Conflict of Interest Statement

The authors declare that the research was conducted in the absence of any commercial or financial relationships that could be construed as a potential conflict of interest.
