# Supplementary material for: Diversity in olfactory bulb size in birds reflects allometry, ecology, and phylogeny
Source: Front Neuroanat. 2015 Jul 29;9:102. doi: 10.3389/fnana.2015.00102 (PMC4518324; doi:10.3389/fnana.2015.00102)
Supplement: Supplementary file 1 [file Table_1.DOCX]

Supplemental table 1. List of species used in this study and volumes (in mm^3^) of their brain, telencephalon (Tel) and olfactory bulbs (OB).

| **Species** | **Order** | **Common name** | **n** | **OB (mm3)** | **Tel (mm3)** | **Brain (mm3)** | **Source** |
| --- | --- | --- | --- | --- | --- | --- | --- |
| *Anas americana* | Anseriformes | American wigeon | 1 | 32.05 | 2778.96 | 4397.88 | This study |
| *Anas castanea* | Anseriformes | Chestnut teal | 1 | 21.72 | 2889.19 | 4366.80 | This study |
| *Anas clypeata* | Anseriformes | Northern shoveler | 2 | 22.74 | 2195.76 | 3310.09 | This study |
| *Anas discors* | Anseriformes | Blue-winged teal | 1 | 17.76 | 1865.93 | 2895.75 | This study |
| *Anas platyrhynchos* | Anseriformes | Mallard | 4 | 33.64 | 4386.68 | 6338.32 | This study, Boire, 1989 |
| *Anas superciliosa* | Anseriformes | Australian black duck | 1 | 36.87 | 3442.08 | 4973.94 | This study |
| *Anser anser* | Anseriformes | Greylag goose | 8 | 58.82 | 7828.35 | 11893.22 | Ebinger and Lohmer, 1987 |
| *Aythya affinis* | Anseriformes | Lesser scaup | 3 | 28.84 | 3095.14 | 4724.65 | This study |
| *Aythya americana* | Anseriformes | Redhead | 2 | 29.68 | 3717.71 | 5525.77 | This study |
| *Aythya collaris* | Anseriformes | Ring-necked duck | 1 | 25.92 | 3149.71 | 4652.51 | This study |
| *Bucephala albeola* | Anseriformes | Bufflehead | 2 | 21.96 | 2393.44 | 3673.79 | This study |
| *Bucephala clangula* | Anseriformes | Common goldeneye | 2 | 30.19 | 3718.39 | 5851.35 | This study |
| *Chenonetta jubata* | Anseriformes | Australian wood duck | 1 | 40.54 | 3127.32 | 4697.88 | This study |
| *Mergus serrator* | Anseriformes | Red-breasted merganser | 2 | 23.65 | 3244.79 | 4915.15 | This study |
| *Tadorna variegata* | Anseriformes | Paradise shelduck | 3 | 23.18 | 2689.60 | 4157.38 | Corfield et al., 2012 |
| *Apus apus* | Apodiformes | Common swift | 1 | 1.36 | 438.14 | 754.25 | This study |
| *Chaetura pelagica* | Apodiformes | Chimney swift | 1 | 1.09 | 159.92 | 342.66 | Boire, 1989 |
| *Collocalia troglodytes* | Apodiformes | Pygmy swiftlet | 1 | 0.59 | 46.40 | 103.68 | This study |
| *Archilochus colubris* | Apodiformes | Ruby-throated hummingbird | 1 | 0.61 | 135.28 | 250.99 | This study |
| *Phaethornis superciliosus* | Apodiformes | Long-tailed hermit | 1 | 0.15 | 96.70 | 189.25 | This study |
| *Caprimulgus sp.* | Caprimulgiformes | Caprimulgus sp. | 1 | 3.19 | 342.75 | 733.59 | Boire, 1989 |
| *Eurostopodus argus* | Caprimulgiformes | Spotted nightjar | 1 | 3.23 | 291.58 | 1141.36 | This study |
| *Charadrius vociferus* | Charadriiformes | Killdeer | 1 | 2.17 | 523.69 | 1073.36 | Boire, 1989 |
| *Haematopus finschi* | Charadriiformes | South Island oystercatcher | 2 | 7.87 | 1757.44 | 2917.70 | This study, Cunningham et al., 2013 |
| *Himantopus himantopus* | Charadriiformes | Black-winged stilt | 1 | 2.91 | 998.82 | 1678.49 | This study, Cunningham et al., 2013 |
| *Larus novaehollandiae* | Charadriiformes | Silver gull | 1 | 2.42 | 720.01 | 2975.65 | This study |
| *Limnodromus griseus* | Charadriiformes | Short-billed dowitcher | 1 | 2.65 | 725.11 | 1123.55 | Boire, 1989 |
| *Limosa lapponica* | Charadriiformes | Bar tailed godwit | 2 | 3.01 | 1563.49 | 2417.27 | Corfield et al., 2012 |
| *Scolopax rusticola* | Charadriiformes | Eurasian woodcock | 2 | 14.71 | 1433.52 | 2338.60 | This study |
| *Sterna hirundo* | Charadriiformes | Common tern | 1 | 4.49 | 808.53 | 1592.66 | Boire, 1989 |
| *Vanellus chilensis* | Charadriiformes | Southern lapwing | 1 | 6.89 | 1686.79 | 2461.39 | Pistone et al., 2002 |
| *Vanellus miles* | Charadriiformes | Masked lapwing | 3 | 8.17 | 1185.57 | 2067.13 | Corfield et al., 2012 |
| *Columba leucomela* | Columbiformes | White-headed pigeon | 1 | 6.74 | 1006.12 | 2355.21 | This study |
| *Columba livia* | Columbiformes | Pigeon | 10 | 7.86 | 1059.84 | 2055.08 | This study, Ebinger and Lohmer, 1987 |
| *Geopelia humeralis* | Columbiformes | Bar-shouldered dove | 1 | 3.20 | 543.29 | 1106.18 | This study |
| *Phaps elegans* | Columbiformes | Brush bronzewing | 1 | 7.46 | 652.25 | 1517.37 | This study |
| *Streptopelia chinensis* | Columbiformes | Spotted dove | 1 | 5.01 | 686.13 | 1430.50 | This study |
| *Streptopelia risoria* | Columbiformes | Barbary dove | 1 | 5.40 | 630.98 | 1140.93 | Boire, 1989 |

| *Dacelo novaeguineae* | Coraciiformes | Laughing kookaburra | 1 | 5.11 | 2451.75 | 3970.08 | This study |
| --- | --- | --- | --- | --- | --- | --- | --- |
| *Haliaeetus leucogaster* | Falconiformes or Accipitriformes | White-bellied sea eagle | 1 | 18.57 | 7456.09 | 11749.03 | This study |
| *Bonasa umbellus* | Galliformes | Ruffed grouse | 3 | 4.99 | 1221.32 | 2288.12 | This study |
| *Callipepla californic* | Galliformes | California quail | 1 | 0.84 | 549.86 | 1007.84 | This study, Cunningham et al., 2013 |
| *Chrysolophus pictus* | Galliformes | Golden pheasant | 1 | 2.21 | 1726.01 | 3368.73 | Boire, 1989 |
| *Colinus virginianus* | Galliformes | Northern bobwhite | 1 | 1.11 | 569.85 | 1090.73 | Boire, 1989 |
| *Coturnix coturnix* | Galliformes | Common quail | 11 | 1.29 | 394.25 | 844.35 | Boire, 1989, Rehkamper et al., 1991 |
| *Coturnix japonica* | Galliformes | Japanese quail | 2 | 3.58 | 419.09 | 846.35 | This study |
| *Falcipennis canadensis* | Galliformes | Spruce grouse | 3 | 2.83 | 1111.40 | 2146.62 | This study |
| *Gallus domesticus* | Galliformes | Chicken | 1 | 3.58 | 1242.46 | 2889.00 | Boire, 1989 |
| *Meleagris gallopavo* | Galliformes | Turkey | 7 | 6.36 | 3269.02 | 6284.93 | This study, Boire, 1989, Corfield et al., 2012 |
| *Numida meleagris* | Galliformes | Helmeted guineafowl | 1 | 1.99 | 2223.28 | 3950.77 | Boire, 1989 |
| *Ortalis canicollis* | Galliformes | Chaco chachalaca | 1 | 9.10 | 1829.65 | 3373.55 | Boire, 1989 |
| *Pavo cristatus* | Galliformes | Indian peafowl | 5 | 9.65 | 2945.92 | 4977.51 | This study, Corfield et al., 2012 |
| *Perdix perdix* | Galliformes | Grey partridge | 14 | 0.78 | 946.07 | 1682.55 | This study, Rehkamper et al., 1991 |
| *Phasianus colchicus* | Galliformes | Ring-necked pheasant | 14 | 7.50 | 1803.12 | 3286.44 | This study, Boire, 1989, Rehkamper et al., 1991 |
| *Tympanuchus phasianellus* | Galliformes | Sharp-tailed grouse | 1 | 1.90 | 1103.20 | 2204.91 | This study |
| *Ardeotis australis* | Gruiformes | Australian bustard | 1 | 41.36 | 6377.92 | 10500.97 | This study |
| *Fulica armillata* | Gruiformes | Red-gartered coot | 1 | 34.61 | 2738.46 | 4015.45 | Carezzano and Bee De Speroni, 1995 |
| *Gallinula tenebrosa* | Gruiformes | Dusky moorhen | 1 | 20.24 | 1652.54 | 2726.54 | This study |
| *Porphyrio porphyrio* | Gruiformes | Pukeko | 3 | 35.22 | 2771.03 | 4186.05 | Corfield et al., 2012 |
| *Acanthorhynchus tenuirostris* | Passeriformes | Eastern spinebill | 1 | 0.31 | 294.40 | 489.38 | This study |
| *Agelaius phoeniceus* | Passeriformes | Red-winged blackbird | 1 | 0.93 | 635.95 | 945.36 | This study |
| *Baeolophus bicolor* | Passeriformes | Tifted titmouse | 2 | 0.42 | 534.22 | 919.53 | This study |
| *Carduelis tristis* | Passeriformes | American goldfinch | 1 | 0.40 | 253.08 | 384.58 | This study |
| *Corvus corone* | Passeriformes | Carrion crow | 12 | 2.00 | 7093.26 | 9477.88 | Rehkamper et al., 1991, Mehlhorn et al., 2010 |
| *Corvus moneduloides* | Passeriformes | New Caledonian crow | 5 | 1.43 | 5558.55 | 7295.40 | Mehlhorn et al., 2010 |
| *Dumetella carolinensis* | Passeriformes | Gray catbird | 1 | 1.48 | 528.68 | 839.35 | This study |
| *Erythrura gouldiae* | Passeriformes | Gouldian finch | 1 | 0.37 | 273.74 | 472.97 | This study |
| *Euphagus carolinus* | Passeriformes | Rusty blackbird | 1 | 1.53 | 1060.44 | 1656.56 | This study |
| *Garrulus glandarius* | Passeriformes | Eurasian jay | 5 | 1.05 | 2571.00 | 3770.82 | Rehkamper et al., 1991, Mehlhorn et al., 2010 |
| *Grallina cyanoleuca* | Passeriformes | Magpie-lark | 1 | 1.82 | 1038.98 | 1668.92 | This study |
| *Cracticus tibicen* | Passeriformes | Australian magpie | 4 | 0.82 | 3443.98 | 4664.24 | Corfield et al., 2012 |
| *Haemorhous mexicanus* | Passeriformes | House finch | 2 | 0.39 | 462.58 | 747.28 | This study |
| *Junco hyemalis* | Passeriformes | Dark-eye junco | 3 | 0.83 | 361.77 | 596.06 | This study |
| *Lichenostomus penicillatus* | Passeriformes | White-plumed honeyeater | 1 | 0.53 | 602.91 | 916.99 | This study |
| *Manorina melanocephala* | Passeriformes | Noisy miner | 1 | 1.03 | 1505.49 | 2278.96 | This study |
| *Melospiza melodia* | Passeriformes | Song sparrow | 3 | 1.09 | 467.26 | 742.86 | This study |
| *Pardalotus punctatus* | Passeriformes | Spotted pardalote | 1 | 0.06 | 186.34 | 400.58 | This study |
| *Passer domesticus* | Passeriformes | House sparrow | 9 | 0.46 | 608.60 | 926.38 | This study, Rehkamper et al., 1991, Mehlhorn et al., 2010 |
| *Passerina cyanea* | Passeriformes | Inigo bunting | 1 | 1.01 | 314.79 | 495.68 | This study |
| *Petroica multicolor* | Passeriformes | Pacific robin | 1 | 0.45 | 266.07 | 473.94 | This study |
| *Poecile carolinensis* | Passeriformes | Carolina chickadee | 1 | 0.10 | 312.22 | 479.65 | This study |
| *Pyrrhula pyrrhula* | Passeriformes | Eurasian bullfinch | 2 | 0.14 | 519.09 | 899.61 | This study |
| *Sitta carolinensis* | Passeriformes | White-breasted nuthatch | 1 | 0.09 | 612.77 | 1000.00 | This study |
| *Spizella passerina* | Passeriformes | Field sparrow | 4 | 0.71 | 280.60 | 472.78 | This study |
| *Spizella pusilla* | Passeriformes | Chipping sparrow | 2 | 0.48 | 245.79 | 396.80 | This study |
| *Stagonopleura guttata* | Passeriformes | Diamond firetail | 1 | 0.54 | 375.81 | 720.08 | This study |
| *Taeniopygia bichenovii* | Passeriformes | Double-barred finch | 1 | 0.18 | 228.40 | 409.27 | This study |
| *Taeniopygia guttata* | Passeriformes | Zebra finch | 1 | 0.13 | 207.83 | 328.19 | Boire, 1989 |
| *Troglodytes aedon* | Passeriformes | House wren | 3 | 0.51 | 292.89 | 462.13 | This study |
| *Turdus merula* | Passeriformes | Common blackbird | 1 | 3.05 | 1208.87 | 1914.09 | This study |
| *Turdus migratorius* | Passeriformes | American robin | 1 | 3.05 | 1135.32 | 1709.38 | This study |
| *Zonotrichia albicollis* | Passeriformes | White-throated sparrow | 1 | 1.17 | 563.80 | 864.35 | This study |
| *Ardea cinerea* | Pelecaniformes | Grey heron | 1 | 18.11 | 5028.04 | 8445.95 | Boire, 1989 |
| *Egretta thula* | Pelecaniformes | Snowy egret | 1 | 9.64 | 1973.35 | 3612.26 | Carezzano and Bee De Speroni, 1995 |
| *Nycticorax caledonicus* | Pelecaniformes | Nankeen night heron | 1 | 6.84 | 1625.14 | 3360.04 | This study |
| *Indicator minor* | Piciformes | Lesser honeyguide | 3 | 2.58 | 325.51 | 587.33 | This study |
| *Indicator variegatus* | Piciformes | Scaly-throated honeyguide | 1 | 4.84 | 332.20 | 597.53 | This study |
| *Melanerpes carolinus* | Piciformes | Red-bellied woodpecker | 2 | 9.36 | 3180.40 | 4160.00 | This study |
| *Melanerpes erythrocephalus* | Piciformes | Red-headed woodpecker | 3 | 10.14 | 2515.73 | 3280.00 | This study |
| *Picoides pubescens* | Piciformes | Downy woodpecker | 1 | 2.00 | 698.83 | 997.53 | This study |
| *Picoides villosus* | Piciformes | Hairy woodpecker | 1 | 15.84 | 3947.10 | 5250.00 | This study |
| *Pogoniulus bilineatus* | Piciformes | Yellow-rumped tinkerbird | 1 | 0.47 | 130.67 | 244.28 | This study |
| *Sphyrapicus varius* | Piciformes | Yellow-bellied sapsucker | 1 | 4.66 | 696.91 | 934.57 | This study |
| *Rollandia rolland* | Podicipediformes | White-tufted grebe | 1 | 13.34 | 1183.89 | 2059.17 | Carezzano and Bee De Speroni, 1995 |
| *Puffinus tenuirostris* | Procellariiformes | Short-tailed shearwater | 1 | 56.09 | 2164.22 | 4592.40 | This study |
| *Thalassarche melanophrys* | Procellariiformes | Black-browed albatross | 1 | 139.26 | 7552.96 | 14129.34 | This study |
| *Alisterus scapularis* | Psittaciformes | Australian king parrot | 1 | 3.72 | 2938.63 | 4394.00 | This study |
| *Aratinga acuticaudata* | Psittaciformes | Blue-crowned parakeet | 1 | 3.13 | 4325.91 | 5410.00 | Fernandez et al., 1997 |
| *Cacatua galerita* | Psittaciformes | Sulphur-crested cockatoo | 1 | 2.50 | 6474.04 | 8142.10 | This study |
| *Eolophus roseicapilla* | Psittaciformes | Galah | 1 | 3.38 | 5520.90 | 6666.15 | This study |
| *Melopsittacus undulatus* | Psittaciformes | Budgerigar | 1 | 0.79 | 825.12 | 1177.61 | Boire, 1989 |
| *Myiopsitta monachus* | Psittaciformes | Monk parakeet | 1 | 4.44 | 2733.13 | 3830.00 | Fernandez et al., 1997 |
| *Pionus menstruus* | Psittaciformes | Blue-headed parrot | 1 | 2.88 | 3851.82 | 5282.82 | Boire, 1989 |
| *Platycercus elegans* | Psittaciformes | Crimson rosella | 1 | 4.16 | 2966.40 | 4013.28 | This study |
| *Platycercus eximius* | Psittaciformes | Eastern rosella | 4 | 1.97 | 2032.15 | 2685.74 | Corfield et al., 2012 |
| *Psephotus haematonotus* | Psittaciformes | Red-rumped parrot | 1 | 2.52 | 1181.44 | 1705.84 | This study |
| *Strigops habroptilus* | Psittaciformes | Kakapo | 1 | 13.07 | 10075.42 | 11979.93 | This study, Gsell, 2012 |
| *Trichoglossus haematodus* | Psittaciformes | Rainbow lorikeet | 1 | 3.66 | 2459.34 | 3301.16 | This study |
| *Eudyptula minor* | Sphenisciformes | Little penguin | 1 | 4.90 | 4338.36 | 7473.94 | This study |
| *Spheniscus magellanicus* | Sphenisciformes | Magellanic penguin | 1 | 31.48 | 10890.21 | 16756.76 | Boire, 1989 |
| *Aegolius acadicus* | Strigiformes | Northern saw-whet owl | 1 | 3.22 | 2009.90 | 3142.86 | This study |
| *Athene cunicularia* | Strigiformes | Burrowing owl | 1 | 15.28 | 4813.80 | 6090.00 | Alma and Bee De Speroni, 1992 |
| *Bubo scandiacus* | Strigiformes | Snowy owl | 1 | 9.35 | 13921.62 | 18127.41 | This study |
| *Tyto alba* | Strigiformes | Barn owl | 1 | 8.71 | 4108.76 | 5849.81 | This study |
| *Apteryx mantelli* | Struthioniformes | North Island brown kiwi | 2 | 80.76 | 4267.72 | 5298.95 | Corfield et al., 2012 |
| *Dromaius novaehollandiae* | Struthioniformes | Emu | 1 | 217.63 | 13695.99 | 21829.88 | Corfield et al., 2012 |
| *Rhea americana* | Struthioniformes | Rhea | 1 | 59.67 | 10281.31 | 19227.80 | Boire, 1989 |
| *Struthio camelus* | Struthioniformes | Ostrich | 1 | 66.52 | 17984.78 | 27006.26 | This study, Cunningham et al., 2013 |
| *Phalacrocorax auritus* | Suliformes | Double-crested cormorant | 1 | 8.03 | 4341.73 | 7323.36 | Boire, 1989 |
| *Nothura darwinii* | Tinamiformes | Darwin’s nothura | 1 | 2.77 | 809.09 | 1482.37 | Corfield et al., 2012 |
| *Rhynchotus rufescens* | Tinamiformes | Red-winged tinamou | 2 | 8.63 | 1838.21 | 3195.65 | This study, Boire, 1989, Cunningham et al., 2013 |
| *Tinamus major peruvianus* | Tinamiformes | Great tinamous | 1 | 13.87 | 1221.88 | 2242.13 | Corfield et al., 2012 |
| *Amazilia tzacatl* | Trochiliformes | Rufous-tailed hummingbird | 1 | 0.29 | 81.26 | 158.01 | This study |
| *Selasphorus rufus* | Trochiliformes | Rufous hummingbird | 1 | 0.31 | 77.52 | 151.64 | This study |
